# Exploring the Role of Large Language Models in Primary Care: Qualitative Study of Physicians in the United States and the Netherlands

**DOI:** 10.2196/91652

**Published:** 2026-07-10

**Authors:** Ilse Super, Harinder Bawa, Onur Asan

**Affiliations:** 1 Department of Systems Engineering Stevens Institute of Technology Hoboken, NJ United States; 2 Department of Internal Medicine Hackensack University Medical Center Hackensack, NJ United States

**Keywords:** LLM, large language model, primary care, communication, teamwork, diagnostic assistance

## Abstract

**Background:**

Primary care is becoming increasingly complex, with primary care physicians (PCPs) facing rising workloads driven by workforce shortages, growing administrative demands, and expanding clinical responsibilities. Recent advances in large language models (LLMs) offer new opportunities to support PCPs across clinical, administrative, and communication-related tasks within their workflows. Understanding how these technologies are perceived and used in primary care practice is, therefore, critical to inform their safe, effective, and human-centered implementation.

**Objective:**

This study aimed to explore Dutch and US PCPs’ perceptions and experiences regarding the use of LLMs in clinical practice, with particular attention to clinical usability, communication and teamwork, and implications for everyday workflows.

**Methods:**

We conducted a qualitative study using semistructured interviews with 15 PCPs from the United States and the Netherlands. Data were collected between February and June 2025 and analyzed using reflexive inductive thematic analysis.

**Results:**

Ten themes emerged related to the use of LLMs in primary care clinical practice, each theme consisting of a set of subthemes. We found that LLMs are being integrated into primary care as both clinical and communication support tools, assisting with diagnostic reasoning, administrative tasks, workload management, and interprofessional and patient communication. While PCPs reported perceived benefits, they also expressed concerns related to safety, efficiency, authenticity, and the preservation of the therapeutic relationship, highlighting the need for careful and context-sensitive use.

**Conclusions:**

Our findings suggest that LLMs are already being integrated into primary care in diverse ways, with their value shaped by both contextual factors and clinician judgment. Understanding how clinicians navigate LLM use in everyday practice is essential to ensuring that LLMs support high-quality, patient-centered primary care and inform organizational policy and LLM design.

## Introduction

Primary care physicians (PCPs), also referred to as family doctors or general practitioners (GPs), are the cornerstone of the health care system, serving as the initial point of access for patients and facilitating comprehensive care throughout the care process. They manage a broad spectrum of health needs and focus on prevention, diagnosis, and treatment of common conditions, while also dealing with a wide range of other responsibilities, including administrative documentation, quality reporting, and diagnostic research [1]. PCPs also play a central role in coordinating care across disciplines. Effective teamwork with nurses, specialists, and other professionals is crucial in the diagnostic process and ensures smooth care transitions, shared decision-making, and safe information flow [2]. Simultaneously, strong communication and shared decision-making with patients are at the core of primary care, depending on trust, empathy, and mutual understanding.

Primary care systems across the United States and Europe are facing a common challenge of increasing care complexity, administrative burden, and workforce shortages, placing a growing strain on frontline physicians [3,4]. Within Europe, the Netherlands is frequently considered a leading example of a strong, gatekeeping primary care system with high levels of digital maturity, making it a relevant comparator to the United States despite differences in health care organization [5,6]. While the United States operates within a more fragmented, performance-based system and European systems tend to be more coordinated and welfare-based, physicians in both contexts encounter similar pressures related to workload, time constraints, and managing complex patient needs [7]. Increased workload is associated with job stress, burnout, and early retirement among physicians, patterns that are increasingly documented in global primary care research [3,8].

In 2022, OpenAI introduced ChatGPT, a publicly available and scientifically informed tool that demonstrated remarkable capabilities in areas such as decision-making, diagnosis, symptom assessment, and triage [9]. Despite its strong performance, ChatGPT is trained on broad, general data and lacks the domain-specific knowledge required for medical accuracy [10]. Since then, numerous large language model (LLM)–based tools have emerged for primary care. While these models can generate coherent text, pass professional examinations, and sometimes outperform humans, they also raise concerns about hallucination, privacy, security, and bias [10,11]. Many LLMs are publicly available, including ChatGPT (OpenAI), Gemini (Google), Phi-3 (Microsoft), OpenEvidence, and Perplexity, which have not been officially authorized for clinical use in practice. Despite that, a study among GPs in the United Kingdom found that a significant proportion (20%) use these tools in their practice [12].

For the successful and effective implementation of innovative technologies, it is essential to establish user trust and usability by engaging and understanding end users during human-computer interactions [13]. The use of LLMs could potentially be beneficial for primary care doctors by supporting process efficiency, quality of care, and clinical decision-making, but if not carefully managed, it may be a significant burden to the already overdemanded physicians and could lead to unsafe practice [14,15]. It is essential to address the challenges and limitations of LLMs and create awareness among doctors to prevent overreliance and medical errors [10].

In digitally advanced health care environments, both the United States and countries such as the Netherlands are increasingly exposed to emerging technologies, including LLMs. While there has been research on the potential applications and limitations that may affect usability in primary care and clinical practice [11,16], limited attention has been given to how PCPs perceive and experience their use in everyday practice, particularly across different health care contexts. To address this gap, this study explores PCPs’ perceptions and experiences with LLMs in the United States and the Netherlands, focusing on clinical usability, communication and teamwork, and integration into routine workflows. By examining these perspectives across 2 distinct yet comparable Western health care systems, this study aims to generate insights that can inform the responsible and effective integration of LLMs in primary care and support shared learning across settings facing similar challenges.

## Methods

### Ethical Considerations

This study was reviewed and approved by the institutional review board (IRB) of Stevens Institute of Technology (IRB ID 2024-070 (N)). Participants were informed of their right to withdraw at any time without consequence, and oral informed consent was obtained prior to the commencement of each interview. They received a modest incentive for their time, which was not contingent on completion of the interview. All data were anonymized and stored securely to protect participants’ confidentiality.

### Research Design and Participants

This study used a qualitative research design using interviews to explore participants’ experiences, perspectives, and practices related to the use of LLM tools in primary care, and followed the COREQ (Consolidated Criteria for Reporting Qualitative Research) guidelines for qualitative research (Multimedia Appendix 1) [17]. A qualitative approach was chosen to allow for in-depth exploration of complex and context-dependent phenomena and to capture the nuanced views of participants in their own words [18]. Participants were recruited through purposive sampling to ensure that they had relevant experience and insight into the topic under investigation and were informed by maximum variation principles to capture perspectives across 2 comparable yet structurally distinct primary care systems and practices [19]. We also used snowball sampling, which occurred when participating PCPs shared the study invitation with colleagues within their professional networks. Inclusion criteria required participants to be a PCP (MD), either in the United States or the Netherlands, be fluent in English, and have experience with an LLM tool in their practice. Most PCPs from the United States were recruited through the research team’s personal network and subsequent referrals. One PCP from the United States was approached independently through LinkedIn. All Dutch PCPs were recruited through national GP associations (“Arts en Zorg” and “Landelijke Huisartsen Vereniging”) and subsequently through referrals. Every PCP was approached and given information about the interview through email. During the interviews, participants described working in a variety of practice contexts and settings (urban or rural and individual practice or large organization). However, organizational size and rural or urban classifications were not systematically collected or formally assessed as part of the study. Informed consent was obtained prior to data collection. An incentive of a US $30 gift card (€30 [US $34.80] gift card for Dutch participants) was provided for each participant after interview completion.

### Data Collection

Semistructured interviews were conducted by the lead author (IS). No prior relationship was established with participants, although the research advisor was acquainted with some. Participants were informed about the researcher’s role, research aims, and broader PhD topic. The interviewer has an interest in LLMs, holding a generally positive view while acknowledging associated risks, and a broader research focus on teamwork and communication in primary care. Data were collected via Zoom (Zoom Communications, Inc), a widely used videoconferencing platform that supports audio communication and automated transcription. The data collection took place from February to June 2025. An interview guide (Multimedia Appendix 2) was used to ensure consistency across interviews while allowing flexibility to follow up on relevant themes that emerged during the conversation and was pilot-tested with 1 physician. The questions covered demographic information and questions regarding the research aim. Demographics, such as years of experience as a PCP, were self-reported by participants during the interviews and therefore reflect perceived professional experience rather than verified employment records. Similarly, other demographic and background characteristics, including gender, nationality, affinity with IT, and tools used in practice, were collected through self-report during the interviews rather than through a separate structured survey instrument. Participants were given the opportunity to provide additional input at the end of the interviews. All interviews were audio-recorded via Zoom with participants’ consent. Video was not recorded, as IRB approval permitted the collection of audio data only and did not allow for analysis of nonverbal communication. Interview sessions lasted approximately 30-45 minutes. The audio recordings were automatically transcribed using Zoom’s built-in transcription feature. Subsequently, all interview transcripts were manually reviewed for accuracy by listening to the audio recordings and verifying the corresponding textual data. Transcripts were anonymized and were not returned to participants for review.

### Thematical Analysis

Interview transcripts were analyzed using reflexive thematic analysis, following an inductive, data-driven approach described by Virginia Braun and Victoria Clarke [18,20], covering the 6 steps of data familiarization, generation of initial codes, searching for themes, reviewing themes, defining and naming themes, and producing the final report. The analysis aimed to provide a rich thematic description of the dataset, appropriate for an underexplored area, capturing patterns of meanings across participants’ experiences with LLM use in clinical practice. The study was conducted within a realist (essentialist) epistemological framework, assuming that participants’ accounts reflect their experiences and perspectives in a direct and meaningful way. Coding was conducted manually in a Microsoft Excel spreadsheet, and themes were identified and defined as patterns of shared meaning across codes relevant to the research questions and were reviewed to ensure that they accurately represented the dataset. Themes were identified at a semantic level, focusing on the explicit content of participants’ accounts, without extending into deeper latent interpretation. We coded as many interviews as were needed to reach data saturation [20]. Data saturation was assessed iteratively during data collection and analysis and was considered achieved when no new codes or themes emerged from successive interviews and when additional data did not contribute further to the development or refinement of existing thematic structure (N=15) [21]. All initial analyses were conducted by the lead author (IS). To enhance the rigor of the analysis, the lead author developed an initial codebook using affinity diagramming, engaged in regular reflexive discussions with an experienced advisor (OA) and practicing PCP (HB), and code checking with 2 assisting bachelor students (BG and AK), who are acknowledged for their contributions. Themes were presented with supporting participant quotations to ensure consistency between data and findings, with both major and minor themes clearly reported.

## Results

### Participant Characteristics

On average, the interviews lasted 32 minutes and 9 seconds, with the shortest interview lasting approximately 18 minutes and the longest interview approximately 47 minutes. Our sample comprised 15 PCPs, including 8 (53.3%) from the Netherlands and 7 (46.7%) from the United States. PCPs used a mix of publicly accessible LLMs (eg, ChatGPT, Perplexity, and OpenEvidence) and integrated LLMs, mostly artificial intelligence (AI) scribes (eg, Juvoly and Abridge). About 33% (5/15) of the PCPs reported to have a significant role in IT within their practice (eg, chief medical information officer) or possessed specialized knowledge on IT. Table 1 presents additional demographic data collected during the interviews, covering gender, PCP type, experience in the profession, LLM tools mentioned during the interview, and their affinity expressed with IT.

Through inductive thematic analysis, we revealed 10 primary themes related to the use of LLMs in PCP clinical practice. Each theme covers a set of subthemes. Multimedia Appendix 3 presents an overview of all themes and subthemes, including their definitions. Multimedia Appendix 4 provides additional representative quotes for each subtheme.

**Table 1:** Participant demographics.

PCP^a^ ID	Sex	Country	Type of PCP	Experience (years) as PCP	Tools mentioned	Affinity with IT
PCP 1	Female	United States	Internal medicine and pediatrics	6	ChatGPT	—^b^
PCP 2	Female	United States	Family medicine and geriatrics	20	AI^c^ scribe and ChatGPT	— ^b^
PCP 3	Male	Netherlands	General practitioner	31	ChatGPT, Perplexity, PO, Mistral, and OurMind	Chief medical information officer
PCP 4	Female	Netherlands	General practitioner	6	AI scribe, ChatGPT, and EvidenceHunt	Chief medical information officer
PCP 5	Male	United States	Family medicine	11	OpenEvidence and ChatGPT	—^b^
PCP 6	Male	Netherlands	General practitioner	20	Juvoly	—^b^
PCP 7	Male	United States	Internal medicine	18	Doximity GPT, OpenEvidence, and Decks Microsoft (ambient listening)	Chief digital officer
PCP 8	Male	Netherlands	General practitioner	29	ChatGPT	—^b^
PCP 9	Male	Netherlands	General practitioner	6	Notebook Google	Chief medical information officer
PCP10	Male	Netherlands	General practitioner	12	Juvoly and ChatGPT	—^b^
PCP 11	Female	United States	Family medicine resident	<1	OpenEvidence	—^b^
PCP 12	Female	Netherlands	General practitioner	1	AI scribe, ChatGPT, and Gemini	PhD in eHealth
PCP 13	Female	United States	Family medicine	16	Abrige, OpenEvidence, Microsoft Co-pilot, and ECG AI tool	—^b^
PCP 14	Male	Netherlands	General practitioner	5	OurMind and Ask Aletta	—^b^
PCP 15	Male	United States	Family medicine	17	ChatGPT, OpenEvidence, and AI Scribe	—^b^

^a^PCP: primary care physician.

^b^Not available.

^c^AI: artificial intelligence.

### Theme 1: Diagnostic Assistance

Our interview data showed that a frequently used case of LLMs in primary care practice was assistance with differential diagnosis. It was helpful for PCPs for complex, difficult, or rare cases, or when they felt that something was missing in their assessment. The LLM tool helped them figure out what was going on with the patient, while still maintaining their own interpretation of the patient’s symptoms. A PCP stated an illustrative example:

I had a person in front of me a few months ago, a lady, 60 years old, really, really sick. I was sure, I'm going to send her to the hospital, but I was not having a single clue what was going on. So with her consent, I was able to prompt an LLM [...]. What is your diagnosis? And bizarrely it ended up with a really rare systemic disease which I afterwards looked up in my guidebook, and it was right.

PCP 9, NL

LLMs also showed to be a good alternative or a useful addition to specialist assistance. LLMs provided the PCPs with specialist knowledge on a wide range of diagnoses that they do not possess themselves or cannot easily access. PCPs describe the LLMs as quicker and more easily accessible, such as this PCP:

...otherwise I would have called my specialist colleague from the hospital nearby, or I would have needed another, [...] sending a request or a question to the medical specialist from the nearby hospital, and then, with a delay of a couple of days I will get my answer back. And now I can get to my suggestions straight away.

PCP 14, NL

To draw medical conclusions, PCPs often did individual research to build up a rapport for patient cases. LLMs were described as functioning as an assistive search engine for tasks ranging from looking up simple definitions to gathering complex scientific research. PCPs appreciate the broad range of knowledge that LLMs can quickly access and provide to them in the right context. A PCP said:

And I really like that, of course, because we as family medicine doctors, there are so many topics we need to know a lot about, of course, but we don’t. That's just not feasible. So it's also really nice to kind of stay up to date but also frame that in a way that is primary care based.

PCP 13, US

Another use case described by PCPs was interpreting medical test results, which can sometimes be difficult for PCPs because they may contain unexpected findings (eg, blood work and magnetic resonance imaging reports). Using LLMs to provide additional information on results was described by a PCP:

So if I’m having some lab results, and I cannot quite figure out why the blood is different than what I would think, in combination with the symptoms, and sometimes I type in the symptoms with the physical examination and the lab results, and then I also ask them to give some interpretation, just to see if I’m also missing out on something.

PCP 12, NL

When using LLMs for diagnostic assistance and decision-making, PCPs pointed out the ability of LLMs to prevent bias, such as availability bias and anchoring bias, by providing evidence-based reasoning behind their suggestions and providing a second opinion. LLMs supported their thought process and reminded them of potential missed information, as described by this PCP:

The benefit for me is, I might undercover uncover a different diagnosis or a consideration. The other thing that I'm interested in through these models is a little bit of the reasoning. Why might one thing be more likely than another? And I might be seeing what I could learn from thinking through that. I'm just kind of interested in the clinical reasoning, because I know that there are certain cognitive biases that clinicians have in terms of information availability.

PCP 7, US

### Theme 2: Streamlining Routine Tasks

PCPs described using LLMs for streamlining routine tasks, such as using them for simple calculations, such as blood pressure or medication dosage.

For instance, when a patient has a note with all his blood pressures written on it, then it’s kind of chaotic. Then I send the note anonymous to perplexity and say, can you calculate the mean blood pressure.

PCP 3, NL

PCPs also expressed their liking for LLMs for summarization of a consultation after using an AI scribe to document the conversation, translating audio transcripts into usable language for documentation.

But now the AI scribe, that is just quite revolutionary. So, it is basically listening to the whole conversation, so, all you may be doing is just putting the orders in, but it's basically listening to your conversation between you and the patient, and it summarizes and transcribes the entire note.

PCP 2, US

Additionally, it is also reported to be used for professional writing and administrative tasks, for example, writing appeal letters and doctors’ notes for patients, and creating vacancies and policies for the practice.

I find it most useful in terms of, drafting an appeal letter to an insurance company for an imaging study that was denied. And I basically inputted, give me a draft email of how to write the appeal letter, and for these reasons, without putting in specific patient information, and it gave me a 3-paragraph letter that was nicely worded.

PCP 1, US

One PCP also described how LLMs can be specifically used for writing narrative history of present illness data.

I guess even narrative HPI data and then having the large language model prioritize or identify some key issues or things that really need to be addressed or worked on.

PCP 5, US

### Theme 3: Workload Support

Another emerging theme that PCPs were experiencing was the use of LLMs for workload support. Many PCPs experienced time savings when having LLMs assist with documentation and research. They described having to search for information scattered over the internet in different databases and protocols as a time-consuming and sometimes difficult task that can be quickly done by an LLM tool, which does a thorough job and provides evidence safely.

It’s saves me a lot of time in administration, because I don't have to Google and search and find the right kind of articles. I don't have to go on PubMed. It basically presents all the suitable research already for me. So, it saves me time.

PCP 4, NL

Another way in which PCPs described being supported in their workload is that it helped them with efficiency. PCPs emphasized how they did more work within a consultation or treated more patients with LLM assistance in the same amount of time: “Well, by saving time I can take more patients in my practice” [PCP 6, NL]. In addition, some also described that the tool can reduce cognitive load and burden, describing how LLM assistance (eg, AI scribes) made them feel more relaxed when the LLM made notes during consultation or when it took over tedious tasks: “I still think the number of tasks doesn’t change. It takes some burden of the task of writing down the consult, and that's a very good one” [PCP 3, NL]. Although some PCPs thought that the number of tasks did not change, some thought that it could take over some of the workload of PCPs. They expected LLMs to take over tasks such as writing letters and answering patient questions, which could free up time for physicians:

I think that when I think there are ways that I do see it adding value to the patients in the future, though, for example, if we're using more and more tools to take some of this growing in-basket workload off the physicians, it could start and advance practitioners.

PCP 5, US

### Theme 4: Interprofessional Communication and Teamwork

Some PCPs mentioned that they used LLMs to help them communicate with other health care professionals. They used the tool to improve their team messaging by having an LLM draft an initial message or improve the quality and tone of their message:

I think it’s improved the quality of my communications to the team dramatically. It’s able to help me find a style and tone that’s hopefully going to land. Well, especially in these non-face-to-face communications like, email, where it’s really easy to misconstrue tone if you’re not really careful, so I think it’s led to a lot of improvement.

PCP 5, US

Another way in which LLMs improved communication between health care professionals was by bridging the gap in expertise between disciplines, supporting collaboration and shared decision-making:

I think it kind of bridges our disciplines. Because it can help us, like, with cardiology and the AI ECG tool. [...] because of this transdisciplinary and a collaborative tool, when you come to your colleague in cardiovascular, you say, well, the tool told me this, and these are the symptoms. Can you please help me and advise how to proceed? It’s a simple example, of course, but I feel when it’s supported by both departments, it can really help also in communicating the results.

PCP 13, US

Another more administrative way LLMs also supported communication between health care professionals was by taking automated discussion notes from recorded professional meetings:

Communication with fellow health professionals, I would say, maybe by having some intervention or meetings with colleagues where minutes were taken by the system. So again, the recording part.

PCP 14, NL

### Theme 5: Patient-Centered Communication

PCPs highlighted the use of LLMs to improve patient-centered communication. Similar to professional use for team communication, they described using LLMs to provide patient-friendly messages and answer questions:

Yeah, I’ve been indeed, but this I think the first and single time I’ve been using it, ask Aletta, when I really was wondering how to reply to a patient with an e-consultation and a digital request in my inbox. So, I asked Aletta, I got a response, I shaped the response or made it a bit more patient friendly language, and then I replied back. So yeah, the doctor with actually the system replied. But I edited a bit.

PCP 14, NL

PCPs also used LLMs for simplifying clinical information for patients. Medical jargon is not always easy for patients to comprehend, especially when receiving test results. LLMs can be helpful in translating medical texts into plain language that is easy to understand. This also made health care more accessible for patients with low literacy:

...sometimes it gets difficult to be able to communicate some of that with patients who don’t have the medical knowledge, or medical literacy, that we do, so in some ways I could see it being helpful in kind of, taking all that medical jargon and making it a lot more colloquial or understandable to someone who didn’t go through all the training that we did.

PCP 11, US

Some PCPs also talked about how AI scribes specifically helped by restoring contact between patient and provider. They emphasized how it took focus away from taking notes and looking at a screen to paying more attention to the patient, allowing for more patient-centered care:

...with the AI listening with you, if you don’t have to type anything in, your concentration on the patient is better. You make more eye contact, you can see more of the verbal and nonverbal communication which the patient sends, you can be more polite by your posture towards the patient.

PCP 6, NL

Considering the use of LLMs in communication toward the patients, PCPs also mentioned that the output of LLMs was often more empathic and patient-friendly:

...the answer that is formulated by the AI is a little bit more. How should I say? User friendly? It has a much friendlier tone, it can be like a little bit more empathetic sounding, whereas a nurse may just say, okay, I’m sorry to hear that, can you tell me these questions, but the AI may go into more details, so it kind of helps them, but not always.

PCP 2, US

Another way in which PCPs have seen useful integration of LLMs in patient-centered care was using it for shared decision-making between the doctor and the patient, as the LLM presented useful information and specialist knowledge during discussions:

I mean definitely, I can see a lot of opportunity for it in shared decision making so as a tool to augment the discussion between a physician and a patient by presenting timely and appropriate information and data.

PCP 5, US

PCPs also mentioned using it for after-visit summaries to provide information to the patient after consultation. AI scribes and other LLM tools can create summaries based on the consultation, helping the doctor and the patient by not missing out any important information:

Then if you end up at the doctor, you might use those LLMs to make summaries and to make summaries that are good for doctors and good for the patients themselves, since we all know people tend to forget stuff when they leave the consultation. So, there are so many benefits there to improve the full experience for a doctor and for a patient perspective.

PCP 9, NL

### Theme 6: Caution With LLM for Communication

Although PCPs mentioned many positive aspects of using LLMs in their communication, they also talked about caution being taken when doing so. One of the things PCPs said was that they were cautious about losing personal touch, as the vocabulary used was different from their personal language, which could alter the tone of the message. They also described that messages become less unique and less personal:

Honestly, the way that it writes sometimes is a little...it’s not as conversational as I would like it to be, and I think it adds a lot of words that aren’t normally used in everyday language. So I think that it just wouldn’t give me the tone that I’m looking for a lot of the time.

PCP 11, US

Other reasons for PCPs not to use it for communication efforts were wanting to keep short ties with their colleagues and patients. They mentioned that it did not improve their efficiency when communicating online and a lot of communication could be done in real time or in person: *“*If I communicate with another provider, my communication is usually like 15 to 30 second email or chat message. And the reply probably is just as efficient” (PCP 15, US). PCPs who do use LLMs for communication often validate their output before sending it off, as LLM-created drafts could introduce errors or misconstrued meaning. They also described validating and editing to make sure that the right tone was used or to give it more of a personal touch before sending:

I never share a broad communication that comes out of ChatGPT, without having reread it to make sure that I'm comfortable with the way that it’s being presented. So, I always read it back over, because sometimes it will change in the attempt to kind of improve that style, it’ll change a meaning accidentally by how the words are arranged, and so I’ll have to do some subtle editing.

PCP 5, US

### Theme 7: Patient Looking up Symptoms

PCPs are not the only people in the consultation room who use LLM tools; patients also use some publicly available LLMs. Some PCPs reported that they knew or heard patients using LLMs to look up their symptoms, and some viewed it as an alternative to Google, which has been widely used by patients in the past to look up symptoms:

I’m not afraid of it. As I am not afraid of Google. People look up a lot by Google, but I know well, I should know my stuff and my profession, so I’m still able to say to patients, well, that’s good information, I think you’re right, or it’s wrong information, maybe, better do this or that, because I think it's better for you, and it’s more to the guidelines.

PCP 6, NL

Not every PCP was enthusiastic for patients to use LLMs in their care process, but some did express their enthusiasm and thought that LLMs will be a useful addition for patients to use. They thought that patients could use it to better understand clinical information, to create an understanding of their symptoms and diagnosis, and provide reasoning and urgency in their communication toward physicians:

And I think what you’re going to see is that people will start with LLMs as part of their care journeys. And you know, do I need to go to the doctor or not will be something that will be increasingly available, I think, being able to translate the complex jargon and words of research papers, and CT Scans and doctor’s notes into something that is easier to understand and grasp, I think, will be really revolutionary. So, I think a lot of it’s going to be on the patient, direct end of it.

PCP 7, US

While it could be a useful addition, some other PCPs talked about careful considerations when patients use the LLMs, as it could introduce anxiety when they misinterpret medical information or could oversimplify based on the information provided to the LLM. With this, it becomes apparent that the level of knowledge and information provided by the patient determines the quality and trustworthiness of the model output:

...you have to be very careful how you ask how you denounce your symptoms, and how you write it down, and you have to be very careful what model you use and how trustworthy it is so, yes, there are risks, but I think the benefits will be more.

PCP 3, NL

### Theme 8: Model Limitations and Concerns

PCPs also talked about the limitations and concerns regarding the models themselves. Generally, PCPs were aware of hallucinations and took caution when using LLMs and validating the output. Catching hallucinations can be tricky for PCPs, and missing incorrect output can cause safety issues, which in this case gave one PCP a paranoid feeling:

No, I’m still a little bit cautious about it, because I know there are hallucinations. I know it can sometimes give an answer that looks correct, but isn’t correct, so I’m still a little bit paranoid.

PCP 3, NL

PCPs also said to be aware of the fact that LLMs are built on data-bound intelligence, meaning the model is trained on broad data and a mathematical model: “The model is only as good as the information data that it’s trained on. So, I think there’s a risk” (PCP 7, US). Another limitation mentioned by PCPs of the LLM models is the lack of traceability and reproducibility, as it can be difficult to retrace old interactions and provide inconsistent answers over multiple uses:

It does, but there’s going to be no way for me to like...I’m not going to record those references for future purposes, whereas, like a textbook or up to date, I can always go back to go back there and find those references if I wanted.

PCP 15, US

### Theme 9: Ensuring Validity

One way to overcome some of the limitations and concerns with LLMs was to ensure the validity of the output before using it. One way PCPs shared how they did this was by manually double-checking based on personal knowledge, gut feeling, rereading, or challenging the model by asking additional questions. Although output is perceived to be mostly accurate, double-checking by challenging the model and critically thinking about the output provided helped the doctors to maintain the quality of their health care provision:

...that’s also something that you have to validate as a doctor. And I think for that, you just need your own smart brain. Yeah, a little bit blunt. You have to be sharp about it and think, okay, this is like a very suitable answer. It looks right. I can use this one. Or this is a very weird answer. I can’t use it. So you have to use your own gut to see that.

PCP 4, NL

PCPs also mentioned validating by source reliability as an additional way to validate the output. Often, LLMs provided references included in their output, which could be validated by looking at the reliability of the source and what methods were used. Checking the references provided by the LLMs assured doctors of the quality and accuracy of the LLM output and gave them a sense of security:

And that’s why I use perplexity, perplexity gives the sources, and then I can check which sources it uses. Sometimes I see it uses a source like, for instance, MedNet, which is not a reliable source, and then I am a little bit suspicious about the answer.

PCP 3, NL

In addition to checking the model’s output, some PCPs also highlight the importance of prompt writing and input. When looking for very specific content or questions, input provided to the model (eg, questions, documents, and information) was a big contributor to the output of the model. This was also specifically commonly mentioned among the Dutch PCPs, as a lot of information LLMs are trained on is based on American standards, and if they wanted to look up Dutch standards, they had to clearly specify this in their prompt to get the right information: *“*I’ve learned the hard way, providing more information gives me much better results than giving little information” (PCP 8, NL).

### Theme 10: Patient Safety and Data Security

The final theme was patient safety and data security. Most of the PCPs mentioned concerns about data storage and breaches. They highlighted that many LLMs store information and that there are no laws or regulations specifically addressing data handling for LLMs, which is especially important in the health care context:

I think there needs to be some sort of rules or regulations from a safety perspective because, let’s say I’m being very cautious about not putting in any information but if other folks are putting information, I’m not entirely sure if it’s true, but like it probably gets built into some sort of large database that I imagine is probably hard to retrieve back from, or take it down from.

PCP 1, US

This is also related to some PCPs who talked about trust in technology providers. A lot of trust is put into the hands of large companies and their integrity, which some PCPs perceived as a risk. Especially, as LLMs are widely adopted in the health care industry, it is important to ensure quality, safety, and accessibility:

I think that that that it and the trustworthiness of an app needs to be really forward, that is one of the most and important aspects, and I do have these feelings about like Juvoly. But I have to admit that I didn’t do really a background check or so to this app. But I think that the big app, that ChatGPT, it’s more like who owns ChatGPT. What happens with the data? It’s more dangerous, I think, it seems more dangerous for us.

PCP 10, NL

The way PCPs tried to prevent data breaches and ensure patient privacy was deidentification and anonymization of the patient data they provided to the LLM, which was especially important when using public LLMs that are not integrated into the electronic health record (EHR) to remain Health Insurance Portability and Accountability Act (HIPAA) compliant: “When it comes to privacy, you just have to be really careful about not filling out like a date of birth, or a name, or whatsoever. I don’t think people do it, but you never know” (PCP 12, NL). Many PCPs mentioned the limited system integration as a security barrier. They struggled with transferring information safely from the EHR to separate systems. EHR integration could be a way to make LLM use more regulated and controlled, which is important to ensure privacy and patient safety:

So ChatGPT is not integrated in our EHR. It’s a separate thing. So, I have to be careful when I put information, ChatGPT. I have not used it for my medical profession on a regular basis, but if I do, I have to be extra careful, like making sure that all the information is HIPAA compliant. And all the patient identifiers are removed and we’re just asking for pure information...

PCP 2, US

One PCP brought up the issue of liability, describing how traditional methods such as textbooks seem more reliable than a new tool, as they remain accountable for their care delivery:

I mean, you can imagine like, if we were in like a courtroom or something, and you were on one side and I was on the other side as a physician, and you had gotten harmed by something that I did. And my answer to your lawyer was like, Why did you do this? And my answer was, you know, ChatGPT told me to do it versus my answer was, well, here’s the textbook. You know the textbook says to do this thing that I did. and it didn’t work out for you, but it’s still standard practice, according to the textbook that pretty much the majority of like us physician’s use. You’d have some sympathy for that answer right?

PCP 15, US

LLMs have the potential to help PCPs, but it was also essential for them to maintain patients’ trust. PCPs talked about the perception of patients when using LLMs and making sure that they did not lose their trust, as they could be perceived as less professional or make the patient feel uncomfortable by using it. Patients losing trust due to reasons such as harmful recommendations and privacy concerns could eventually cause patients to distrust their provider, which could harm the relationship between the doctor and the patient:

So, safety in terms of protecting their health is one perspective, but also safety in in the perspective of safeguarding the biggest importance, most important value in healthcare. That’s trust. If someone does not trust me anymore, because it seems like a recorder is on the background, pumping all my data to some other country where they have opinions about me. Then I might lose this trust. And then I can’t do my work properly. So yeah, it is really really important to be aware of that to do an informed consent?

PCP 9, NL

PCPs also highlighted the risk of overreliance on LLM outputs, which could pose a safety issue for both the PCP and the patient. This could occur when taking over recommendations or statements that are incorrect due to overtrusting the LLM to be correct:

And one of the concerns that I have again, with the Large language model in that setting is that they could make recommendations or statements that aren’t actually like medical best practice, and that it could easily be missed because people just get in the habit of, you know, hitting.

PCP 5, US

## Discussion

### Principal Findings

This study provides an in-depth qualitative examination of how 15 Dutch and American PCPs integrate LLMs into clinical practice. The findings demonstrate that LLMs are being used as clinical support tools, enhancing diagnostic reasoning, streamlining administrative tasks, and relieving (cognitive) workload for PCPs. Next to that, they are identified as multifaceted communication support tools that facilitate interactions and teamwork among PCPs, specialists, and patients, and, in some cases, informally supplement specialist consultation when access is limited. At the same time, PCPs articulated important concerns related to safety, data security, efficiency, authenticity, and the preservation of the therapeutic relationship, underscoring the need for careful, context-sensitive use. To our knowledge, this is the first paper to highlight key aspects of LLM use in primary care settings, directly from PCPs in the United States and the Netherlands. The qualitative design of this study allows for the firsthand perspectives of PCPs to be captured and shared, based on real experiences. It is interesting to note that PCPs in 2 different countries had similar thoughts and concerns regarding the use of LLMs in primary care, as both countries were represented along all identified themes.

### Comparison With Prior Work

LLMs are rapidly emerging as powerful tools across many areas of medicine, including primary care [22]. As the foundation of preventive health and the first point of contact for most patients, PCPs often operate under significant time pressures and limited resources [23]. As a result, LLMs are being actively explored and increasingly implemented in primary care practices to help clinicians manage workload and improve the efficiency and quality of patient care [24].

Physicians undergo years of training in medical school and residency to build a strong medical knowledge base initially and then, with experience, refine this knowledge to learn illness scripts that guide their diagnostic reasoning. This is especially important when caring for patients with complex conditions or atypical presentations, where subtle cues and pattern recognition are essential for accurate diagnosis. Several PCPs discuss their methods of using LLMs in assisting their diagnostic reasoning. For example, some clinicians may use LLMs to develop a differential diagnosis and use them to double-check that no important possible diagnoses have been overlooked. PCPs offered an interesting perspective, noting that LLMs can be used as a safeguard against cognitive biases, such as anchoring and availability bias, by prompting clinicians to reconsider initial impressions and broaden their diagnostic thinking. Although many of these tools were not vetted for this use, other PCPs in the United Kingdom have also reported using them for this purpose [12].

Another key area in which LLMs can assist PCPs is in rapidly synthesizing information at the point of care. This spans the whole clinical workflow—before, during, and after a visit. Prior to the visit, physicians often prechart by reviewing prior notes, specialist consultations, hospital records, and relevant laboratory and imaging results. Several PCPs highlighted that LLMs can be used to summarize prior data and interpret complex results. For the complex patient, precharting also involves digging into literature to find treatment recommendations and weighing all the risks and benefits. One PCP highlights that LLMs can condense this work from hours to minutes while probably being more comprehensive in their review. During the visit, PCPs may need to quickly reference current guidelines or evidence-based recommendations to inform treatment plans. LLMs may be used to summarize data that patients bring in, such as blood pressure or blood glucose logs. Clear and concise patient instructions are also written for patients as a point of reference in most cases, and LLMs can do this quickly as well. After the visit, clinicians must integrate all this information into a clear, accurate, and concise note. Other administrative tasks include reviewing results and communicating them to the clinical team and patients, reviewing medication refill requests, and writing appeal letters for medications or imaging tests. LLMs can help streamline each of these steps, which are also reported as opportunities throughout the literature [12].

One major area of development worth mentioning is the use of LLMs to synthesize a comprehensive and accurate clinical note at the end of a patient visit [25]. AI scribes or ambient voice technology automatically captures clinical conversations and has been shown to decrease documentation time by 28.8%, alleviating physician burnout [26]. Physicians report feeling less distracted and more connected with the patient during the visit itself. Several of the interviewed PCPs highlight this aspect of LLM-listening technologies, which are already being implemented and have gotten support from GPs in European primary care [14,27]. Reducing technological workload by engaging with computers during consultations may also enhance patient satisfaction with the clinical encounter [28].

Communication and teamwork emerged as central aspects of LLM use in primary care. Participants described LLMs as facilitating bidirectional communication among PCPs, specialists, and patients, positioning these tools as quasi-members of the health care team, a vision that previous research among PCPs found for the consultation of the future [29]. Within interprofessional collaboration, PCPs reported using LLMs to support team messaging and to bridge communication gaps across specialties and, in some cases, as an informal substitute for specialist consultation when questions were perceived as low risk. In patient-facing communication, LLMs were used to draft responses to patient messages, simplify clinical information, support shared decision-making, and enhance empathetic tone. Despite these perceived benefits, PCPs expressed concerns about potential loss of personal connection, efficiency trade-offs, and the risk of errors, emphasizing the need for careful validation before patient-facing use. Participants also reported mixed views on patients’ use of LLMs for health-related communication: some welcomed increased patient autonomy, while others expressed caution regarding misinformation and its impact on the doctor-patient relationship. Previous studies found that most American patients look for health information on the internet, and 1 study even showed that for 81% of their respondents, the information provided by ChatGPT is at least as useful as the information provided by their doctor [30,31]. Patients communicating information they found online can have a serious effect on the doctor-patient relationship, especially if the information brought up is deemed inaccurate, and, therefore, it is important to understand how PCPs respond to patients communicating LLM-generated findings and how they can support patients in finding accurate information [32].

PCPs also highlight some of the risks in using LLMs for such tasks. First, patient privacy and data security are important factors and appear to be the primary concerns among PCPs. Concerns are raised regarding how information that is input into an LLM is used and stored. Second, LLMs have been noted to have hallucinations, or instances where the output has been fabricated by the LLM itself. This requires users to proofread each output and ensure its accuracy. Output depends on the resources that the LLM reviewed, and so results need to be validated to ensure that it is correct medical information. Third, some PCPs expressed concern about overreliance on automated documentation. If clinicians begin to depend too heavily on AI-generated notes, critical thinking and active information processing during the visit may decrease over time. The shortfalls of LLMs have been widely discussed in the literature, and the most important point for PCPs is to be conscious and aware of these shortfalls and use effective ways to overcome or prevent the negative aspects of the tools, for example, by not inputting HIPAA-protected information and validating outcomes to prevent patient harm [33-35]. Additionally, when PCPs notice potential risk of harm for the patient or low quality of output, they are likely to not use the AI service in the future [36].

### Implications for Practice

The findings suggest several implications for practice across design, workflow, policy, and training. PCPs described concerns regarding hallucinations, overreliance, transparency, and data security when using LLMs and reported developing their own strategies, such as validating outputs, assessing source reliability, and using prompt engineering, to mitigate these risks. These clinician-driven practices highlight opportunities for tool developers to integrate safeguards directly into system design, reducing reliance on individual work-arounds and supporting safer, more trustworthy use of AI [37]. At the workflow level, LLMs were perceived as valuable for supporting workload by saving time, reducing cognitive burden, and assisting with administrative and routine tasks, suggesting that prioritizing tools that align with these needs may improve efficiency while allowing greater focus on direct patient care. At the organizational level, the reported use of publicly accessible LLMs for clinical purposes underscores the need for clear, context-specific policies that address appropriate use, data security, and accountability, particularly as technological advances outpace broader regulatory frameworks. In addition, the findings indicate a need for targeted training, as PCPs reported using LLMs beyond their originally intended purposes. One university is already offering a combined doctor of medicine and master of science in AI degree, which is one step on the way to PCPs prepared for a future involving regular LLM use [33]. Training efforts should address both the potential benefits and the risks of LLM use, including strategies for effective validation and communication, to support responsible integration while preserving clinician judgment and maintaining patient trust.

### Limitations and Further Research

Some limitations of this study include the small sample of physicians from only 2 countries. Although the in-depth analysis of the interviews provided rich insights and perspectives, the limited sample size could restrict the generalizability of our findings to broader populations of PCPs. Future research could validate certain findings and insights with larger sample sizes and qualitative or mixed method designs for strengthened generalizability. This study captures early-stage perceptions of LLM use rather than outcomes from in-context clinical trials; however, such insights are critical for informing the design and evaluation of future rigorously trialed applications within real-world primary care settings [38]. Further studies may be conducted to see whether LLM use improves diagnostic accuracy and, therefore, overall mortality. Other studies may be conducted to look at efficiency in seeing patients and whether this improves health care access, changes in patient satisfaction, and improvements in physician well-being. Additionally, the expanding role of LLMs will likely reshape medical education, opening a large area of investigation into how trainees learn diagnostic reasoning, documentation, and communication skills from the beginning of their training. With thoughtful implementation and continued research, LLMs can become a powerful aid in the work of PCPs.

### Conclusions

Our findings suggest that LLMs are already being integrated into primary care in diverse ways, with their value shaped by both contextual factors and clinician judgment. By highlighting both the advantages and disadvantages of LLM-supported clinical practice and communication, this study contributes to a more nuanced understanding of how these technologies are reshaping primary care in everyday clinical practice. As LLMs become increasingly embedded in primary care, understanding how clinicians navigate their use in real-world practice will be essential to ensuring that these tools enhance, rather than undermine, high-quality, patient-centered care.

## Appendix

Multimedia Appendix 1 COREQ checklist.

Multimedia Appendix 2 Interview guide.

Multimedia Appendix 3 Themes, subthemes, and definition per subtheme.

Multimedia Appendix 4 Themes, subthemes, and representative quotes per subtheme for each category.

## Abbreviations

AI: artificial intelligence
COREQ: Consolidated Criteria for Reporting Qualitative Research
EHR: electronic health record
GP: general practitioner
HIPAA: Health Insurance Portability and Accountability Act
IRB: institutional review board
LLM: large language model
PCP: primary care physician

## Data Availability

The qualitative interview data generated and analyzed during this study are not publicly available due to confidentiality and privacy considerations inherent to interview-based data with health care professionals. Deidentified excerpts may be made available from the corresponding author on reasonable request.
