# Supplementary material for: Exploring the Role of Large Language Models in Primary Care: Qualitative Study of Physicians in the United States and the Netherlands
Source: JMIR Med Inform. 2026 Jul 10;14:e91652. doi: 10.2196/91652 (PMC13401071; doi:10.2196/91652)
Supplement: Multimedia Appendix 2 [file medinform_v14i1e91652_app2.docx]

# Multimedia Appendix 2

# Interview Guide

## Introduction (Dutch)

- **Bedankt** voor uw bereidheid om deel te nemen aan dit onderzoek.
- Zoals vermeld eerder zal als jij dat geen probleem vindt het interview straks plaats vinden in het **Engels,** maar ik zal eerst even korte introductie geven over mezelf en het onderzoek, en samen door het informed consent gaan.
- Ik zal mezelf en het onderzoeksproject kort voorstellen.
- In de komende 30-45 minuten wil ik graag inzicht krijgen in uw ervaring met een tool gebaseerd op een groot taalmodel, zoals ChatGPT, binnen uw beroep. Alle gedachten en gevoelens op basis van uw ervaring zouden nuttig zijn voor het onderzoek.
- **Doel:** Deze kwalitatieve, verkennende studie richt zich op interviews met huisartsen uit de VS en Europa. Het doel is om te begrijpen hoe zij gebruik maken van producten gebaseerd op grote taalmodellen, zoals ChatGPT, bij hun dagelijkse zorgtaken en teamcommunicatie.
- Er zijn geen goede of foute antwoorden. Het doel is om te begrijpen wat uw ervaring is met deze tools in uw beroep en wat u ervan vindt. Als u ergens geen mening over heeft of geen ervaring mee heeft, kunt u dat gerust aangeven.
- Het interview wordt opgenomen en het gesprek wordt getranscribeerd om de gegevensanalyse te vergemakkelijken. Alle informatie die u verstrekt, wordt vertrouwelijk behandeld en alle identificerende details worden uit het eindrapport verwijderd om uw privacy te beschermen.
- Het interview is vrijwillig. U kunt op elk moment uw toestemming intrekken en stoppen met deelname. U kunt ook weigeren bepaalde vragen te beantwoorden zonder dat dit gevolgen heeft voor uw deelname aan het onderzoek. Welke beslissing u ook neemt, er zijn geen nadelige gevolgen of verlies van voordelen waar u anders recht op had.
- Als u vragen, opmerkingen of zorgen heeft over het onderzoek, kunt u dit op elk moment aangeven. Heeft u op dit moment vragen?
- Dan wil ik nu ook vragen om de camera uit te zetten vanwege privacy beleid rondom het onderzoek en of we het interview verder kunnen voortzetten in het Engels.
- Dan start ik straks de opname en vraag ik nogmaals om toestemming om dit gesprek op te nemen. Dat doe ik omdat ik de toestemming voor het opnemen ook vast wil leggen.

## Introduction (English)

- Thank you for your willingness to participate in this study.
- I will first give a brief introduction about myself and the research and go through the informed consent with you.
- … [I will briefly introduce myself]
- Over the next 30-45 minutes, I would like to gain insight into your experience with a tool based on a large language model, such as ChatGPT, within your profession. Any thoughts and feelings based on your experience would be valuable for this study.
- **Objective:** This qualitative, exploratory study focuses on interviews with primary care physicians from the U.S. and Europe. The goal is to understand how they use large language model-based products, such as ChatGPT, in their daily clinical tasks and team communication.
- There are no right or wrong answers. The aim is to understand your experience with these tools in your profession and your perspective on them. If you do not have an opinion on something or have no experience with it, you can simply indicate that.
- The interview will be recorded, and the conversation will be transcribed to facilitate data analysis. All information you provide will be treated confidentially, and any identifying details will be removed from the final report to protect your privacy.
- Participation in this interview is voluntary. You may withdraw your consent and stop your participation at any time. You may also choose not to answer certain questions without any consequences for your participation in the study. Whatever decision you make, there will be no adverse effects or loss of benefits to which you would otherwise be entitled.
- If you have any questions, comments, or concerns about the study, please feel free to bring them up at any time. Do you have any questions at this moment?
- I would now like to ask you to turn off your camera due to privacy policies related to this study.
- I will now start the recording and ask again for your permission to record this conversation, as I want to document consent for the recording as well.

## Introduction (5 minutes)

- STOP VIDEO
- START RECORDING
- Before we begin, I want to confirm that it's okay for me to record our conversation and that we have gone over the consent form together on which you agree to participate. If that is correct?

## Warm-up questions (5 minutes)

- Could you please introduce yourself
- Could you give a description of your profession and for how long you have been in this position?

## Main Questions (20 – 30 minutes)

**Experience**

- 1. Could you **specify the LLM based tool** or software that you have been using?
     - Is the tool *connected to your EHR system*? Transcription of consultation one year. Medical research/specific questions.
  2. What is you **experience** with the LLM tool?
     - Do you use LLMs in your *personal life*? Barely use them at the moment.
     - How often do you use it?
  3. For **what activities** have you used the tool in your **profession**? (Decision-making process, patient/interprofessional communication, administrative tasks)
     - Probe on:
     - Could you give an **example** of how you have used it?
  4. How would you describe the benefits of using the tool in your profession?
     - **Example** of how you experience these benefits.

**Challenges and concerns**

- 1. What are the **challenges** in your experience of using the tool in your profession?
     - Probe on:
     - How do you *overcome* those challenges?
  2. What do you think about LLM tools with regards to *safety?*
  3. How do you feel about using LLM tools in your profession?
     - Comfortable?
     - Do you *trust* the tool?
  4. How accurate do you think answers provided by the tool are?
  5. How do you validate the outcomes provided by the tool?
  6. What potential ethical concerns do you have regarding the use of LLM tools in primary care?
  7. Are there any regulations currently in place for the use of LLMs
     - If yes, what rules, and what do you think of these rules?
     - If no, do you think there should be? And what should these rules be?

**Workflow**

- 1. How did the tool change the way you work? (change in **workflow)**
     - Do you think that in general, LLM tools create *more additional tasks or does it make your work easier*?
  2. How do LLMs impact your *decision-making process* (in practice)?
  3. How would you describe that LLM tools impacted the way you *provide care to your patients*?
     - Can you tell me how you think LLMs *add value* to the care you provide for your patients?

**Communication**

- 1. How would you describe LLM tools impact teamwork and communication?
     - Probe on:
     - *Fellow healthcare professionals*?
     - *patients*?
  2. How do you *perceive patients to respond* when you use the LLM tool during communication?
  3. Have you ever *experienced patients using LLMs for communicating with you?*
     - *Could you elaborate on that with an example?*

**Future/potential**

- 1. Do you think aspects of your profession will change with the adoption of LLM tools? (change in profession)
  2. Do you think LLM tools could lower your workload? (administration, communication with patient)
  3. Where do you see the potential benefit for patients in the use of LLM tools in your profession?
  4. How do you think patients would respond to the use of the LLM tools in your communication?
  5. Do you think LLM tools will be widely adopted by the healthcare industry, and specifically your profession in the near future?
     - How do you feel about that?
  6. What are the barriers that you perceive that limit the adoption of LLM tools on a wider scale in your profession?
  7. How do you think LLM tools could help improve access to healthcare in underserved populations?
  8. How do you think LLM tools could be used to address health disparities among different populations?

## Conclusion (5 minutes)

- Is there anything else you would like to add that we have not covered?
- Tell about giftcard procedure and if they want to know more about the publication.
- Thank the participant, ask for referral.
