# Supplementary material for: Exploring the Role of Large Language Models in Primary Care: Qualitative Study of Physicians in the United States and the Netherlands
Source: JMIR Med Inform. 2026 Jul 10;14:e91652. doi: 10.2196/91652 (PMC13401071; doi:10.2196/91652)
Supplement: Multimedia Appendix 3 [file medinform_v14i1e91652_app3.docx]

Multimedia Appendix 3

Themes, Subthemes, and Definition per Subtheme.

| **Theme** | **Subtheme** | **Definition** |
| --- | --- | --- |
| **Diagnostic assistance** | Differential diagnosis | Refers to the use of LLM based tools to assist with differential diagnosis (e.g., for complex, difficult or rare cases). |
|  | Specialist assistance | Refers to using LLMs as an alternative to asking a specialist for information or assistance. |
|  | Research and information gathering | Refers to using LLMs as a search engine for tasks ranging from simple definitions to complex scientific research. |
|  | Interpreting test results | Refers to using LLMs for interpreting medical test results (e.g., blood work, MRI reports). |
|  | Bias check | Refers to the use of LLMs to address bias in reasoning and coming to an answer by having it give another look at your thought process and diagnostic options. |
|  |  |  |
| **Streamlining routine tasks** | Simple calculations | Refers to the use of LLM based tools for doing simple calculations (e.g., blood pressure calculations, medication dosage). |
|  | Consultation summarization | Refers to the use of LLM based tools to summarize consultations based on audio transcripts into usable language. |
|  | Professional writing and administration | Refers to the use of LLMs for professional writing (e.g., creating an appeal letter, vacancies, writing policies). |
|  | Narrative HPI data | Refers to the use of LLMs for writing a patient’s chronological development of illnesses or problems, including symptoms and treatment. |
|  |  |  |
| **Workload relief** | Saving time on documentation and research | Refers to the time saved during the day due to the use of LLMs in practice for documentation and research (e.g., AI scribe more efficient than taking consultation notes, database search). |
|  | Increased physician efficiency | Refers to the higher turnover of patients due to more efficient working physician when using LLMs in their work. |
|  | Reduced cognitive load and burden | Refers to the cognitive relief and taking of the burden on difficult or repetitive tasks so the primary care physician has a more relaxed workload (e.g., less typing due to AI scribes). |
|  | Reduced workload | Refers to the reduction of work by having LLM take over part of the workload, potentially solving problems with shortages. |
|  |  |  |
| **Interprofessional teamwork and communication** | Team messaging | Refers to the use of LLMs for writing messages within the healthcare team among professionals. |
|  | Bridge gap between disciplines | Refers to the use of LLMs for bridging the knowledge gap between disciplines and supporting collaboration, making shared decision-making easier. |
|  | Automated discussion notes | Refers to the use of LLMs for recording meetings between professionals and timing the minutes of the discussion. |
|  |  |  |
| **Patient-centered communication** | Patient messages and question answering | Refers to the use of LLMs for messaging to the patient or answering patient questions through online portal. |
|  | Simplifying clinical information | Refers to the use of LLMs for simplifying clinical information to communicate the information to the patient (e.g., simplifying MRI reports). |
|  | Restoring contact between patient and provider | Refers to the perception that LLMs could restore the contact between patient and provider as technology eases parts of the consultation and gives the doctor the ability to focus more on the patient in front of them instead of their notes (e.g., automatic consultation summarization from recordings). |
|  | More Empathic | Refers to the LLM being more empathetic in its writing for patient messages. |
|  | Shared Decision Making | Refers to the use of LLMs for communicating and collaborating with the patient for shared decision making. |
|  | After visit summaries | Refers to the use of LLMs for consultation summaries after a visit which can help both doctor and patient to document everything and make managing care easier. |
|  |  |  |
| **Caution with LLM for communication** | Losing personal touch | Refers to primary care physicians could help with or facilitate communication, but caution is necessary to not lose a personal touch in language. |
|  | Keeping short ties | Refers to limiting the use of LLMs for communication with colleagues as direct real-time communication is quicker and easier and keeps ties short between colleagues. |
|  | Validation before sending to patient | Refers to the risk of sending texts to patients that have not been checked by a professional and highlights the need for validation before sharing or communicating LLM generated text to patients. |
|  |  |  |
|  |  |  |
| **Patients looking up symptoms** | Alternative to Google | Refers to patients using LLMs as an alternative to Google to look up their symptoms. |
|  | Useful addition | Refers to the perception of PCPs on patients using LLMs to look up symptoms and bringing it up during consultation as a useful addition to patient-provider interaction. |
|  | Appropriate for situational and conditional use | Refers to caution advised by PCPs for patients using LLMs to look up symptoms as it is only appropriate in some situations under the right conditions. |
|  | Caution for diagnostic anchoring | Refers to the caution PCPs should have when patients use LLMs to look up symptoms as patients might get biased due to diagnostic anchoring. |
|  |  |  |
| **Model limitations and concerns** | Caution for Hallucinations | Refers to caution during the use of LLMs and validating the output for model hallucination. |
|  | Data-Bounded Intelligence | Refers to the nature of LLMs where it is trained on mostly broad information and remains a mathematical model where inherently the model is only as good as what it is trained on and how its built. |
|  | Lack of traceability and reproducibility | Refers to the lack of retracing old interactions or creating the same interaction each time with the LLM. |
|  |  |  |
| **Validation techniques** | Validating by double checking | Refers to validating the output of the LLM by manually double checking based on personal knowledge, gut feeling, re-reading or challenging the model by asking additional questions. |
|  | Validating LLM source reliability | Refers to validating the output of the LLM by checking the sources provided by the LLM for reliability (e.g., look up the method or study design). |
|  | Importance of prompt writing and model input | Refers to the importance of asking the right question to the LLM and providing the right information to ensure reliable output of the model. |
|  |  |  |
| **Patient safety and data security** | Concerns about data storage and breaches | Refers to fears about unauthorized access and the responsibility and security of storage of sensitive patient information. |
|  | Trust in Technology Providers | Refers to the lack of confidence in vendors, LLM developers, or platforms to handle data responsibly. |
|  | De-identification and Anonymization | Refers to removing identifiable information before using patient data for AI or research. |
|  | System Integration as a Security Barrier | Refers to how disjointed systems and LLMs not being integrated into the EHR can hinder secure use. |
|  | Liability | Refers to PCPs struggling with liability when using LLMs in their care process. |
|  | Maintaining patient trust | Refers to the risk of losing the patients trust due to AI use as this is important for the care process and patient safety. |
|  | Overreliance | Refers to PCPs relying too much on the LLM tool, potentially providing wrongful medical advice or recommendations that might cause patient harm |
