# Supplementary material for: Exploring the Role of Large Language Models in Primary Care: Qualitative Study of Physicians in the United States and the Netherlands
Source: JMIR Med Inform. 2026 Jul 10;14:e91652. doi: 10.2196/91652 (PMC13401071; doi:10.2196/91652)
Supplement: Multimedia Appendix 4 [file medinform_v14i1e91652_app4.docx]

Multimedia Appendix 4

Themes, subthemes, and representative quotes per subtheme for category 1: clinical work support

| **Theme** | **Subtheme** | **Representative quotes** |
| --- | --- | --- |
| **Diagnostic assistance** | Differential diagnosis | 1. Well, let me specify it as a thought co-pilot. I might use it for a difficult case, which I might get once a month, where I really want to sit down and think through a complex case. (PCP 7, US) 2. Sometimes I use it, when I have a feeling that something's off, but I cannot quite figure out what's going on. So, then I put in the symptoms of the patient with my findings and physical examination, and I tell ChatGPT to give me differential diagnostics just to see if I'm missing something out. (PCP 12, NL) |
|  | Specialist assistance | 1. I think, especially in family medicine, you're aware that you don't know as much, as a medical specialist does. And I think these tools kind of provide us a library of knowledge. (PCP 13, US) 2. But just thinking about one of my studies on hemoglobinopathies, for example, we are kind of lacking an institutional guideline for that, like a lot of institutes don't have a good institutional guideline. So when I go back to open evidence, it kind of adds to asking our experts. And it gives me a good idea about what tests I could do or how to refer, or when to refer. So that's kind of helpful. (PCP 13, US) |
|  | Research and information gathering | 1. Sometimes to search in our guidelines what I should do and also sometimes when it's not on our guidelines but it's in our journal, because I know I saw some article about it. Sometimes it's more easily to give it out in ChatGPT than search it on the website, because sometimes the website you need to search it also, and then ChatGPT is quicker in it. So I think those 3 reasons are the are the main reasons I use it in the counseling room. (PCP 12, NL) 2. I've used it for a few small things where something where I knew the answer. but like didn't have the exact answer in my head memorized. but like when you see it, you know it's right or not. So stuff where you can ask a quick question, and you know, when you get the answer, like, you know, you've got the right thing. so nothing like really complicated something really easy, something that I might have used Google for in the past. (PCP 15, US) |
|  | Interpreting test results | 1. Yeah, I mean, sometimes we, we can request CT scans and MRI scans, and sometimes the, the results we get that can be quite complicated. I'm not a radiologist but based on the results and the symptoms of the patient and say that like the ask me of experts, which is our institutional guidelines basically. You can kind of give advice based on that. So that, that really makes it very nice. You know, it really is integrated into our clinical workflow. (PCP 13, US) |
|  | Bias check | 1. The general approach might be to read the textbook article using the up-to-date tool or service. But here I might present a case where I provide a little bit of the history and some of the testing and work up-to-date and might ask it for you know what else you know. Could I be thinking? Or I might play around with it where I might just give it the case and tell it what I might do, one version where I tell it, what I'm thinking I might do, another version where I try to not bias it and leave it and leave it without my thought process in there. I like to think through the case 1st myself, so I'm not relying on that for my thinking, but it's generally a good helpful adjunct, if something doesn't quite add up or make sense. (PCP 7, US) 2. the availability bias, the anchoring bias you kind of coming to the 1st thing that you get or the premature closure. You know scenario where you think thought about something. If it's 3 out of 4 criteria, and then you just kind of accept it, as you know, meeting all 4 criteria. But it doesn't really. So, I'm interested in in this tool as being a bit of a cognitive check on that. And another is like, you know, there may be things that I haven't even considered that I might want to ask or think about, and those could be helpful. (PCP 7, US) |
|  |  |  |
| **Streamlining Routine Tasks** | Simple calculations | 1. I do have one of my assistants. She uses it all the time, and she also uses it, for instance, to calculate an average blood pressure, for instance. (PCP 4, NL) 2. So in the clinic, in the primary care setting, I've used it when I'm looking at dosing for things. So if I need to quickly figure out what medication dose is required for a certain medication, I would use it for that. (PCP 11, US) |
|  | Consultation summarization | 1. I have been using speech recognition. And I think it's also good to mention that I have been working as a data collector for a company that provides speech recognition for primary care. I have been using that for I think over a year now and basically it transcribes my consultations and automatically summarizes that into a suitable GP summary. That's something I've been using as an LLM. (PCP 4, NL) 2. It records, so I use my phone in clinic. I ask, and we use it through Epic, which is our electronic healthcare record. And it's basically, when I come into the room, I ask the patient for their consent for me to use the Abridge and then I start recording, and then it creates a note for me after the visit. (PCP 13, US) |
|  | Professional writing and administration | 1. I just have asked ChatGPT, can you formulate a letter? So, there is no identifiers or anything, just a generic letter. Just so it makes it sound better. And it's also very professional.” (PCP 2, US) 2. I forgot to mention that I haven't used it yet, but will be in the near future, is for drafting and modifying policies. And that's another area, that for clinical policies, it makes it much easier to quickly create one, and then kind of iterate the draft very effectively. (PCP 5, US) |
|  | Narrative HPI data | 1. I guess even narrative HPI data and then having the large language model prioritize or identify some key issues or things that really need to be addressed or worked on. (PCP 5, US) |
|  |  |  |
| **Workload Relief** | Saving time on documentation and research | 1. I don't have to get into databases to see the Dutch protocols on specific diseases, which takes much more time than asking GPT to get the answer for me. In which answer I always see that it goes into the protocol I was looking in otherwise, so it saves me time in that way. It helps me find the right page in a protocol of certain diseases in which I'm not quite certain if I have to go left or right with the patient. So that saves me time and makes it safe. (PCP 8, NL) 2. I used to care for a pretty complex set of patients with postural orthostatic tachycardia syndrome, which is a sort of poorly characterized, difficult to treat condition that has some evidence, but it's kind of scattered and not often easy to find. And so I remember spending evenings, for a period of time, scouring different databases, pulling articles, reading them, and trying to bring them together, and I think I could have taken to 10 hours of work and put them into 20 min with open evidence now, and probably have done a more thorough job of it. (PCP 5, US) |
|  | Increased physician efficiency | 1. But in spaces where you can start to increase physician efficiency, you can start to overcome challenges like workforce, you know. Maybe you can have clinicians seeing more patients now or managing a greater panel. Maybe you can make their visits higher yield and more effective. Maybe you can help overcome difficulty hiring medical assistants or other staff members by taking some of their workload off of them and managing them with a large language model. So all of that improves kind of overall system access specifically to underserved populations. (PCP 5, US) 2. I mean, I'm definitely hoping that it will. It's really challenging seeing somebody, for like they might come in with 3 problems. But they might have like one real problem out of the 3, and then in 20 min. kind of diagnosing and then explaining, and then coming up with a plan and then writing it down and writing a note. So if you can turn 20 min of actual like position, time into maybe an hour's worth of like if the AI can like write a detailed note and also transcribe patient instructions that could be really cool, whereas it might have taken me 15 to 20 min to write a really good note, and then another 10 min to write out some good, patient instructions or find some good ones online. So maybe the scribe can do all that for me. (PCP 15, US) |
|  | Reduced cognitive load and burden | 1. Well, as I said before, it gives me especially a cognitive relief. I think, for instance, with speech recognition, which is a very big one, they say, well, it saves time, so you can see more patients. I don't think that that is the case to be honest, because I see quite a few patients in a day, and that already gives me a lot to think about and a lot to process in my mind and using these LLMs makes it more relaxed to do my consultations, and gives me more time to actually see my patients look at them without being stressed about, okay, I need to really write this down. (PCP 4, NL) 2. I think it's an enhancement like in the LLM it's certainly an enhance of in the in the ambient dictation. It's certainly an enhancement of that. Because I generally have everything kind of done, and it's nice, and I do have to edit, but the editing is generally easier and more favorable to do in terms of the diagnostic work most of the time. (PCP 7, US) |
|  | Reduced workload | 1. But I still think I think there can be done more with it. What I what I told before, when I have to ask for a test or write down specialist letter, I think it can take some tasks of me, so I still see a lot of possibilities to use it in the whole practice. We're just at the beginning, I think. (PCP 3, NL) 2. So right now, everybody has instant answers on their fingertips like, you type in a question on your phone, and you get the answer right away. So the new generation expects that and I see that sometimes, they send us a MyChart message and say, Hey, I sent you a MyChart message. I sent you a message 2 hours ago. You did not responded yet. But they don't realize we're in clinic. We're seeing patients. And we answer these questions between our patients. So our nurses have to keep reminding them. Yes, it's a MyChart message. It's not an urgent portal. We will get back to you whenever we can. So the AI can definitely help meet that demand. (PCP 2, US) |
|  |  |  |
| **Interprofessional teamwork and communication** | Team messaging | 1. I can force my colleagues also to be more structured and use the same format of writing, since in healthcare in the Netherlands these days, it's not like you work full time. You have to work together to take care of your patients, and that is a big advantage with LLMs, you can instruct them. You can force them to incorporate stuff, that does helps a lot. (PCP 9, NL) 2. I think it really helps us frame and kind of give us evidence-based outlines for our messages to patients and colleagues. (PCP 13, US) |
|  | Bridge gap between disciplines | 1. I think that'll be very helpful, because, maybe we can ask ChatGPT to create a message. It'll be nice to say, can you send a message to, so we do a lot of like in basket Med within the Ehr system. I can send a message to my team. I can send a message to anybody in the University of Wisconsin system. So you know, I'm not really sure like I could say that. Say, I see a patient and because I'm a primary care physician, I'm not a specialist, I think it can be helpful, although I do some geriatrics, I do some consultation, and I usually send my notes to the person who has referred that patient to me. But I think, as a specialist like, say, you're a cardiologist, and you want your primary care physician to know about what you thought. So you can just tell once you do your note like, and that AI scribe. (PCP 2, US) 2. I've used it for other professional communication. What I mean by clinical communication is, you know, from a you know, from me to another physician about a patient. (PCP 7, US) |
|  | Automated discussion notes |  |
|  |  |  |
| **Patient-centered communication** | Patient messages and question answering | 1. The second type of communication that we would do with patients is see if they've gotten some blood work and results or something that we need to communicate with the patient. Typically, we'll call the patient. Let them know the results if they are signed up for our messaging, and I guess information there to for their chart with, we call it my chart, where they can communicate with a doctor. We can send them a message through there again. I think that would have to require information. Patient information being put into the model saying that these are the patients results. This is what I'd like to communicate to the patient in terms of, we'll know what to do about these results, and then I'll probably give a paragraph or 2 that can then be communicated to the patient. Alternatively, we could use ChatGPT to generically say, Hey! please describe what a lipid panel is for the patient, and how to decrease the bad cholesterol Ldl, and how to increase the good cholesterol, Hdl, and see if whatever inputs is concise and something generically, we could use for clarification or education. (PCP 1, US) 2. Yeah, very frequently, I think. Especially when we have to send notes to our patients, for example. On certain depth outcomes, or advice we received from colleagues. (PCP 13, US) |
|  | Simplifying clinical information | 1. I've also used it for radiology reports. We request X-rays basically for patients. And we always get a radiology report back which is written down by a radiologist which is incomprehensible for our assistants and our patients. So especially our patients who have basically low vocabulary skills. So I use LLMs anonymously to basically translate that into comprehensible Dutch, so I basically give ChatGPT the prompt to translate it from a radiology report into comprehensible language, and I give it the task to write it down in Dutch level B1 so it's understandable for the patients. (PCP 4, NL) 2. I could see ChatGPT, or other things like that, come up with a teaching plan for explaining this condition to patients like that could be really good. Cause up to date really explains it to physicians, but not to patients. (PCP 15, US) |
|  | Restoring contact between patient and provider | 1. The benefits of the OurMind. Let's say the consultation aid, it restores the contact between the patient and the doctor, because you can just look at the patient and listen and have a conversation without taking care of making notes, because it's all written down and it's it saves some time. (PCP 3, NL) 2. Somehow, yes, I mean, I have to press the button to record, and at the end I check what is recorded. And I think I'm less attached to my computer the moment a patient is sitting in front of me because I'm relying a little bit on the system. Also, it gives me more attention to the person next to me. (PCP 14, NL) |
|  | More Empathic | 1. Now the thing that's different, though, is, the responses from the large language model were much more empathetic. The patients liked the quality of the response is better, but the inaccuracies were a huge problem. (PCP 5, US) |
|  | Shared Decision Making | 1. I really don't feel I refer patients that much to medical specialists at the moment, because I feel I have those AI tools in place. Which really gives me the information to give to the patient, and then, through shared decision-making kind of discuss whether they should be referred or not. (PCP 13, US) |
|  | After visit summaries | 1. Can you write down the summary, and especially the treatment plan in in a way that is comprehensible for patients. So that's an option, that is written down in a very comprehensible way. (PCP 4, NL) 2. My style of notes has been to dictate a note with the patient as long as I'm running on time, and so what I will do is at the end of the visit. I will dictate a note, so that the patient actually, here's a recap of the visits and my assessment and plan. The LLM model would help make that much tighter, much cleaner for a lot of the words, but I still kind of kept my process there now, when I didn't on time then it was nice, because I could still have my note done, and if I was on time, let me go back to. If I was on time I would usually copy and paste that in the my assessment plan into the after visit summary, so that the patient could walk out with a transcript of the note. (PCP 7, US) |
|  |  |  |
| **Caution with LLM for communication** | Losing personal touch | 1. Yes, it does. but there is a big but. We have to make some kind of mixture with our own language and the ChatGPT language, because sometimes I read a post, and I think well, this is ChatGPT. This isn't good, there is no spirit in it, so I think it has to be some something of both. So again, there is a little bit of a threat. but I think in the end it will make communication better. (PCP 3, NL) 2. I think people are, you know, starting to use them to help drafts, memoranda and emails and I would say, for the most part that's welcome. Sometimes you lose the flavor of and that tone of voice that a person uses in lieu of a sanitized LLM version of bullet points. When you leave the verbal and you move into the written and text tone, you lose intonation and things. So there may be a further change to move away from that uniqueness of the message. (PCP 7, US) |
|  | Keeping short ties | 1. And then, in terms of communicating with our colleagues and teams. I'm not entirely sure how that would be helpful, because we're mostly communicating with office staff in regard to appointments when we need to see patients. And that is a decision we're making with the patients after the visit. Or if a patient's calling to see when for a specific problem. Do we need to see them today for an urgent visit tomorrow or within the week? And that's something we're deciding, based on what is the concern. so I don't... that's a decision that I wouldn't leave to ChatGPT or these models to make in real time. (PCP 1, US) |
|  | Validation before sending to patient | 1. I always check that for myself to see if what the ChatGPT writes down, if it's correct or not, and then I'll place it in the patient's file in a way that they can read it themselves. (PCP 4, NL) |
|  |  |  |
|  |  |  |
| **Patients looking up symptoms** | Alternative to Google | 1. Yes, sometimes patients. Mention it, they say. Well, I looked it up on ChatGPT but they there are 2 things. One is I don't think ChatGPT is the right tool to do it, and They don't trust it yet, but well, it's like I said, it will be some time, and then these models get more dedicated to medical questions, and then well, then, they start using it. And I know when patients started Googling. I knew they were Googling. I thought they have to be crazy if they don't Google it. So I start asking them, did you Google it. And then they start turning red and said, Oh, yeah, you don't like that thing. But yes, I Googled it. And then I think at some point 70 % of my patients, Google, their symptoms. But they didn't try. They didn't dare to mention it. And I think that will be the same way with the LLMs. I think, instead of I hear still a lot of people, Googling. But now and then someone says I, I asked it on ChatGPT. (PCP 3, NL) 2. Oh, yes, yes, they have done that. they're usually out in like left field. It's not. It hasn't been any better than like I Googled this question. Because the answers that they come up with are just ridiculous. Usually. (PCP 15, US) |
|  | Useful addition | 1. So this is only a small thing, I observed. But I heard this happening all day with patients doing themselves uploading fool health records to anything, and then figuring out they might have something rare, you know, often are right. So that makes me think like. Oh, I might miss a lot. (PCP 9, NL) 2. Yeah, yeah, there is really increasing that. One patient I've never met before. I mean, he didn't come that often. He came to me with the symptoms. He came to me with the response from ChatGPT. It would have been either diabetes or it was pancreatic cancer. I indeed agree with him that it was a very severe condition what happened? And he was sent to the Internal Medicine Department, but I think he was quite in the right corner. (PCP 14, NL) |
|  | Careful considerations | 1. Yeah. Well, I always tell them best to not do it, because yeah, whether they fill it out in ChatGPT, or whether they fill it out in Google. Perhaps ChatGPT is more accurate. But, like I said, it's that. I think it's important that you have the tools to test it yourself to see if it's reliable information or not. And that's something patients lack of. They do not have the knowledge to know. This is a likely diagnosis I'm having or not, so it can give you some worrying without needing it to be worrying. (PCP 12, NL) 2. Honestly, I don't think the AI generated response was very helpful in that conversation. I think it oversimplified the question. The answer for her. And it kind of it gave her the answer that she was looking for. But the research that I did didn't really corroborate with the AI generated answer that she found. (PCP 11, US) |
|  |  |  |
| **Model Limitations and Concerns** | Caution for Hallucinations | 1. Well, this could reflect a fundamental misunderstanding on my part on how large language models work, but predominantly, these models are taking a lot of different text or information, massive amounts. And from that learning and generating conclusions and the ability to answer something based on what is input to it. And so the quality of what is put into the large language model, or what it's trained on is going to determine its accuracy. And so there's there is the issue of hallucinations which I don't know enough about the technical background to understand if something is only trained on a clinically accurate set of information, whether that still is at risk of hallucinations or not, but for a lot of the models that are out there right now, they are not trained on clinical information, like ChatGPT. (PCP 5, US) 2. Yeah. Well, always before saving it in a patient file, you will have to go through everything thoroughly, but I have a colleague, and I have also heard a little bit of irritation from assistance in the office. Concerning that the wrong data was entered in the wrong file. For example, there's some manual work in it and indeed the hallucination part. It's a bit funny, but the shorter sometimes the conversation is it wants to make up own content. And it's always a little bit similar. So I can recognize also what is written down as it's really crap. And it's not true. (PCP 14, NL) |
|  | Data-Bounded Intelligence | 1. This is trained on a broad swath of information that could be inclusive of inaccurate medical information. And so, if it's incorporating that into its process and into its kind of database of text that it draws from, then it's going to be less likely to make appropriate critical thinking and accurate decision making. (PCP 5, US) 2. Like, I might ask Google, something really simple, like, something that it's hard for a computer to mess up like, what does this nerve do? Or where does this muscle attach or show me a picture of this muscle? You know something like that. Or you get a really quick answer. I might use ChatGPT for that now. But for anything complicated, like what is the best treatment for this condition, or, take fibromyalgia, or something that requires like an antibiotic, what is the right antibiotic for this? I'll still turn to up to date, because I know that there's like a person who has vetted that information versus a computer kind of like analyzing a bunch of different things. (PCP 15, US) |
|  | Lack of traceability and reproducibility | 1. I like the idea that that I can. I can see how a model found the answer. It's still a little bit awkward if you can't if it's a totally black box. I don't like that idea. (PCP 3, NL) 2. I mean, it probably is accurate. 95% of the time. It's just that other 5%. And the fact that you can't if I look back 10 months or a year later, and I'm like. Why did I do that? It's pretty easy to for me to go back and say, well, I know I would have looked it up to date and I can look back, and I can find the article where I would have probably found that answer and reference that article down the road, if somebody's like, why did you do that? I could be like, well, it says right here that this is what you're supposed to do. And at least I can explain myself versus if you say Oh, I looked it up on up to date or on on some other resource, that you then don't have necessary access to like if ChatGPT 6 months later pulls up a different article, and they don't come up with the same answer. Well, then I'm kind of screwed if I made a mistake. (PCP 15, US) |
|  |  |  |
| **Validation techniques** | Validating by double checking | 1. I trust it, but I still have I still have doubts, so I'll reread everything it says, and try to get the answer by making another question on just a different way, and to see if those answers are the same, or trying to get into the database where GPT says it has the answer from. (PCP 8, NL) 2. Of course you'll need to like, scan through and make sure the answers are all correct, but I could see that being a really cool use. (PCP 15, US) |
|  | Validating LLM source reliability | 1. Yeah, but that's the thing, because if I use, for instance, evidence hunt, then I can see that there is evidence there that supports the answer. For instance, the diving example, the evidence hunt will show me what kind of research it used to give me those kind of answers, so I can check the research. But that's just me as a person. But I can see, okay, this is a good validated study, or this is like a very small study, so it's not very good advice, but that's something that I do. (PCP 4, NL) 2. That it can be wrong. There's been a handful of times that it will give me incorrect information, and even sometimes for my own knowledge to test it, I will ask it questions to answers, ask it questions for answers that I know and sometimes they'll give the wrong answer, but I know that I have learned what the correct answer is, so I will sometimes use it, but I always take a look at the articles that it cites, and sometimes try to take a look at those articles myself, to see whether or not it's a credible source. (PCP 11, US) |
|  | Importance of prompt writing and model input | 1. yes, it had some influence on my decision making. But in the same way I normally would look in on a website or something like that. But the way I approach these websites that changes because I ask my AI in perplexity in this case. I ask, for instance, what does the Nhg advise on, let's say vitamin D, or I experiment with it. I ask, I have a patient with a vitamin d of 30. She's 27 years old. What is, according to the Nhg. standard the dose of vitamin D I should give? But then I look it up because I want to check it. But I see most of the time, it's okay. But you have to ask a good problem, because if I just say, What dose should I use? Then it comes with American standards, so I have to ask for the Dutch situation. (PCP 3, NL) 2. Most of the time. I remember what kind of journal it was, so I tell the prompt to search in like "huisarts en wetenschap", for instance. in our case or the wtfg, and then it's more accurate defining it. But if you do not do it, then it gives you some general information from the Internet. (PCP 12, NL) |
|  |  |  |
| **Patient Safety and Data Security** | Concerns about data storage and breaches | 1. Yes, because you always have to be very aware that you don't use any patient information. And that's kind of a struggle, because i'm a CMIO. So I'm very aware of these kinds of things. But my staff like my yeah, my staff and my assistants. They aren't. So I have to really be explicit about how to use this kind of tools. And I think it's human nature, because it is so easy just to just upload a photo or upload a file which has patient information. So I think that's a very big yeah, not struggle, but I think that's a big data breach that we can obviously get by the simple tool. Yeah, it's such an easy tool. So the risk of a data breach is very likely. (PCP 4, NL) 2. But when, whenever colleagues are informed about it, besides enthusiasm, there's always this side note about, okay, but what happens with the data and how safe is this? And who's looking into those data. And I understand those questions. They're really, really important. It's health information, potentially very tricky privacy information. So yeah, I understand those concerns. And I think they need to be met. (PCP 14, NL) |
|  | Trust in Technology Providers | 1. And then there's, I think, a category of ethics around like the sort of beneficence and autonomy of the patient. Are you leaking or providing any information to a company, this large language model that it might use, or it might leak and I think that there have to be approaches and guardrails to set that up so that you're it's the appropriate use of the of the of the technology. (PCP 7, US) 2. It's all about big data and good AI, so that's something. Sometimes a thought I have like, how to keep it accessible, how to keep it working, even though a company goes bankrupt or got bought by some other company. So that's a bit of a worry I have sometimes. (PCP 9, NL) |
|  | De-identification and Anonymization | 1. I have to be extra careful, like making sure that you know all the information. It is hipaa compliant. And you know all the patient identifiers are removed. and we're just asking for just pure information. (PCP 2, US) 2. I don't know for sure. And for that reason I'm really careful about what I upload to the model, so no names, no birth dates and etcetera. (PCP 3, NL) |
|  | System Integration as a Security Barrier | 1. I think the biggest challenges, especially trying to use it within healthcare is definitely the concerns about security. And you know, patient information, that sort of aspect. And, I would say, it's not really integrated into our electronic medical record. So we're seeing all the information for a patient on one side and in one system. And if we want to try to incorporate ChatGPT, it's kind of completely separate and so that, I think is definitely a barrier until they figure out how to incorporate and make it safe and secure. (PCP 1, US) 2. So they have one with an integration into the EHR software we use. And there's a web browser version at the moment I push for the web browser one, because that's having more functionality in my situation, and is easier to grasp on in terms of security, because I can't really oversee how the integration fully works of the EHR. (PCP 9, NL) |
|  | Maintaining patient trust | 1. They may then start seeing physicians as just like technicians are. Maybe not a person who's gone through this much schooling to understand. You know how the human body works, and you know, as someone who really maybe knowledgeable and and kind of have sort of the intellect to figure out what's going on. And they may view. Physician says, Oh, look! You know, they're also just using this tool to figure out what's going on. (PCP 1, NL) 2. I think the other ethical issue that I guess I could think about has more to do with like conflict with patients being uncomfortable with it. And you know what to do in the instance that a patient declines, or to having an AI tool being utilized as part of their visit, and whether we can create the ability to be responsive to that or not. (PCP 5, US) |
|  | Overreliance | 1. Recommendations made that harm a patient, or lead to a patient getting inappropriate or inadequate care. That's kind of the biggest thing, the risk of patient safety for that patient themselves. (PCP 5, US) 2. Yeah, I don't validate it. I'll just take my advances from it and move on. (PCP 8, NL) |

Themes, subthemes, and representative quotes per subtheme for category 2: Teamwork and communication.

| **Theme** | **Subtheme** | **Definition** |
| --- | --- | --- |
| **Interprofessional teamwork and communication** | Team messaging | 1. I can force my colleagues also to be more structured and use the same format of writing, since in healthcare in the Netherlands these days, it's not like you work full time. You have to work together to take care of your patients, and that is a big advantage with LLMs, you can instruct them. You can force them to incorporate stuff, that does helps a lot. (PCP 9, NL) 2. I think it really helps us frame and kind of give us evidence-based outlines for our messages to patients and colleagues. (PCP 13, US) |
|  | Bridge gap between disciplines | 1. I think that'll be very helpful, because, maybe we can ask ChatGPT to create a message. It'll be nice to say, can you send a message to, so we do a lot of like in basket Med within the Ehr system. I can send a message to my team. I can send a message to anybody in the University of Wisconsin system. So you know, I'm not really sure like I could say that. Say, I see a patient and because I'm a primary care physician, I'm not a specialist, I think it can be helpful, although I do some geriatrics, I do some consultation, and I usually send my notes to the person who has referred that patient to me. But I think, as a specialist like, say, you're a cardiologist, and you want your primary care physician to know about what you thought. So you can just tell once you do your note like, and that AI scribe. (PCP 2, US) 2. I've used it for other professional communication. What I mean by clinical communication is, you know, from a you know, from me to another physician about a patient. (PCP 7, US) |
|  | Automated discussion notes |  |
|  |  |  |
| **Patient-centered communication** | Patient messages and question answering | 1. The second type of communication that we would do with patients is see if they've gotten some blood work and results or something that we need to communicate with the patient. Typically, we'll call the patient. Let them know the results if they are signed up for our messaging, and I guess information there to for their chart with, we call it my chart, where they can communicate with a doctor. We can send them a message through there again. I think that would have to require information. Patient information being put into the model saying that these are the patients results. This is what I'd like to communicate to the patient in terms of, we'll know what to do about these results, and then I'll probably give a paragraph or 2 that can then be communicated to the patient. Alternatively, we could use ChatGPT to generically say, Hey! please describe what a lipid panel is for the patient, and how to decrease the bad cholesterol Ldl, and how to increase the good cholesterol, Hdl, and see if whatever inputs is concise and something generically, we could use for clarification or education. (PCP 1, US) 2. Yeah, very frequently, I think. Especially when we have to send notes to our patients, for example. On certain depth outcomes, or advice we received from colleagues. (PCP 13, US) |
|  | Simplifying clinical information | 1. I've also used it for radiology reports. We request X-rays basically for patients. And we always get a radiology report back which is written down by a radiologist which is incomprehensible for our assistants and our patients. So especially our patients who have basically low vocabulary skills. So I use LLMs anonymously to basically translate that into comprehensible Dutch, so I basically give ChatGPT the prompt to translate it from a radiology report into comprehensible language, and I give it the task to write it down in Dutch level B1 so it's understandable for the patients. (PCP 4, NL) 2. I could see ChatGPT, or other things like that, come up with a teaching plan for explaining this condition to patients like that could be really good. Cause up to date really explains it to physicians, but not to patients. (PCP 15, US) |
|  | Restoring contact between patient and provider | 1. The benefits of the OurMind. Let's say the consultation aid, it restores the contact between the patient and the doctor, because you can just look at the patient and listen and have a conversation without taking care of making notes, because it's all written down and it's it saves some time. (PCP 3, NL) 2. Somehow, yes, I mean, I have to press the button to record, and at the end I check what is recorded. And I think I'm less attached to my computer the moment a patient is sitting in front of me because I'm relying a little bit on the system. Also, it gives me more attention to the person next to me. (PCP 14, NL) |
|  | More Empathic | 1. Now the thing that's different, though, is, the responses from the large language model were much more empathetic. The patients liked the quality of the response is better, but the inaccuracies were a huge problem. (PCP 5, US) |
|  | Shared Decision Making | 1. I really don't feel I refer patients that much to medical specialists at the moment, because I feel I have those AI tools in place. Which really gives me the information to give to the patient, and then, through shared decision-making kind of discuss whether they should be referred or not. (PCP 13, US) |
|  | After visit summaries | 1. Can you write down the summary, and especially the treatment plan in in a way that is comprehensible for patients. So that's an option, that is written down in a very comprehensible way. (PCP 4, NL) 2. My style of notes has been to dictate a note with the patient as long as I'm running on time, and so what I will do is at the end of the visit. I will dictate a note, so that the patient actually, here's a recap of the visits and my assessment and plan. The LLM model would help make that much tighter, much cleaner for a lot of the words, but I still kind of kept my process there now, when I didn't on time then it was nice, because I could still have my note done, and if I was on time, let me go back to. If I was on time I would usually copy and paste that in the my assessment plan into the after visit summary, so that the patient could walk out with a transcript of the note. (PCP 7, US) |
|  |  |  |
| **Caution with LLM for communication** | Losing personal touch | 1. Yes, it does. but there is a big but. We have to make some kind of mixture with our own language and the ChatGPT language, because sometimes I read a post, and I think well, this is ChatGPT. This isn't good, there is no spirit in it, so I think it has to be some something of both. So again, there is a little bit of a threat. but I think in the end it will make communication better. (PCP 3, NL) 2. I think people are, you know, starting to use them to help drafts, memoranda and emails and I would say, for the most part that's welcome. Sometimes you lose the flavor of and that tone of voice that a person uses in lieu of a sanitized LLM version of bullet points. When you leave the verbal and you move into the written and text tone, you lose intonation and things. So there may be a further change to move away from that uniqueness of the message. (PCP 7, US) |
|  | Keeping short ties | 1. And then, in terms of communicating with our colleagues and teams. I'm not entirely sure how that would be helpful, because we're mostly communicating with office staff in regard to appointments when we need to see patients. And that is a decision we're making with the patients after the visit. Or if a patient's calling to see when for a specific problem. Do we need to see them today for an urgent visit tomorrow or within the week? And that's something we're deciding, based on what is the concern. so I don't... that's a decision that I wouldn't leave to ChatGPT or these models to make in real time. (PCP 1, US) |
|  | Validation before sending to patient | 1. [1]I always check that for myself to see if what the ChatGPT writes down, if it's correct or not, and then I'll place it in the patient's file in a way that they can read it themselves. (PCP 4, NL) |
|  |  |  |
|  |  |  |
| **Patients looking up symptoms** | Alternative to Google | 1. Yes, sometimes patients. Mention it, they say. Well, I looked it up on ChatGPT but they there are 2 things. One is I don't think ChatGPT is the right tool to do it, and They don't trust it yet, but well, it's like I said, it will be some time, and then these models get more dedicated to medical questions, and then well, then, they start using it. And I know when patients started Googling. I knew they were Googling. I thought they have to be crazy if they don't Google it. So I start asking them, did you Google it. And then they start turning red and said, Oh, yeah, you don't like that thing. But yes, I Googled it. And then I think at some point 70 % of my patients, Google, their symptoms. But they didn't try. They didn't dare to mention it. And I think that will be the same way with the LLMs. I think, instead of I hear still a lot of people, Googling. But now and then someone says I, I asked it on ChatGPT. (PCP 3, NL) 2. Oh, yes, yes, they have done that. they're usually out in like left field. It's not. It hasn't been any better than like I Googled this question. Because the answers that they come up with are just ridiculous. Usually. (PCP 15, US) |
|  | Useful addition | 1. So this is only a small thing, I observed. But I heard this happening all day with patients doing themselves uploading fool health records to anything, and then figuring out they might have something rare, you know, often are right. So that makes me think like. Oh, I might miss a lot. (PCP 9, NL) 2. Yeah, yeah, there is really increasing that. One patient I've never met before. I mean, he didn't come that often. He came to me with the symptoms. He came to me with the response from ChatGPT. It would have been either diabetes or it was pancreatic cancer. I indeed agree with him that it was a very severe condition what happened? And he was sent to the Internal Medicine Department, but I think he was quite in the right corner. (PCP 14, NL) |
|  | Careful considerations | 1. Yeah. Well, I always tell them best to not do it, because yeah, whether they fill it out in ChatGPT, or whether they fill it out in Google. Perhaps ChatGPT is more accurate. But, like I said, it's that. I think it's important that you have the tools to test it yourself to see if it's reliable information or not. And that's something patients lack of. They do not have the knowledge to know. This is a likely diagnosis I'm having or not, so it can give you some worrying without needing it to be worrying. (PCP 12, NL) 2. Honestly, I don't think the AI generated response was very helpful in that conversation. I think it oversimplified the question. The answer for her. And it kind of it gave her the answer that she was looking for. But the research that I did didn't really corroborate with the AI generated answer that she found. (PCP 11, US) |

Themes, subthemes, and representative quotes per subtheme for category 3: Risks and concerns

| **Theme** | **Subtheme** | **Definition** |
| --- | --- | --- |
| **Model Limitations and Concerns** | Caution for Hallucinations | 1. Well, this could reflect a fundamental misunderstanding on my part on how large language models work, but predominantly, these models are taking a lot of different text or information, massive amounts. And from that learning and generating conclusions and the ability to answer something based on what is input to it. And so the quality of what is put into the large language model, or what it's trained on is going to determine its accuracy. And so there's there is the issue of hallucinations which I don't know enough about the technical background to understand if something is only trained on a clinically accurate set of information, whether that still is at risk of hallucinations or not, but for a lot of the models that are out there right now, they are not trained on clinical information, like ChatGPT. (PCP 5, US) 2. Yeah. Well, always before saving it in a patient file, you will have to go through everything thoroughly, but I have a colleague, and I have also heard a little bit of irritation from assistance in the office. Concerning that the wrong data was entered in the wrong file. For example, there's some manual work in it and indeed the hallucination part. It's a bit funny, but the shorter sometimes the conversation is it wants to make up own content. And it's always a little bit similar. So I can recognize also what is written down as it's really crap. And it's not true. (PCP 14, NL) |
|  | Data-Bounded Intelligence | 1. This is trained on a broad swath of information that could be inclusive of inaccurate medical information. And so, if it's incorporating that into its process and into its kind of database of text that it draws from, then it's going to be less likely to make appropriate critical thinking and accurate decision making. (PCP 5, US) 2. Like, I might ask Google, something really simple, like, something that it's hard for a computer to mess up like, what does this nerve do? Or where does this muscle attach or show me a picture of this muscle? You know something like that. Or you get a really quick answer. I might use ChatGPT for that now. But for anything complicated, like what is the best treatment for this condition, or, take fibromyalgia, or something that requires like an antibiotic, what is the right antibiotic for this? I'll still turn to up to date, because I know that there's like a person who has vetted that information versus a computer kind of like analyzing a bunch of different things. (PCP 15, US) |
|  | Lack of traceability and reproducibility | 1. I like the idea that that I can. I can see how a model found the answer. It's still a little bit awkward if you can't if it's a totally black box. I don't like that idea. (PCP 3, NL) 2. I mean, it probably is accurate. 95% of the time. It's just that other 5%. And the fact that you can't if I look back 10 months or a year later, and I'm like. Why did I do that? It's pretty easy to for me to go back and say, well, I know I would have looked it up to date and I can look back, and I can find the article where I would have probably found that answer and reference that article down the road, if somebody's like, why did you do that? I could be like, well, it says right here that this is what you're supposed to do. And at least I can explain myself versus if you say Oh, I looked it up on up to date or on on some other resource, that you then don't have necessary access to like if ChatGPT 6 months later pulls up a different article, and they don't come up with the same answer. Well, then I'm kind of screwed if I made a mistake. (PCP 15, US) |
|  |  |  |
| **Validation techniques** | Validating by double checking | 1. I trust it, but I still have I still have doubts, so I'll reread everything it says, and try to get the answer by making another question on just a different way, and to see if those answers are the same, or trying to get into the database where GPT says it has the answer from. (PCP 8, NL) 2. Of course you'll need to like, scan through and make sure the answers are all correct, but I could see that being a really cool use. (PCP 15, US) |
|  | Validating LLM source reliability | 1. Yeah, but that's the thing, because if I use, for instance, evidence hunt, then I can see that there is evidence there that supports the answer. For instance, the diving example, the evidence hunt will show me what kind of research it used to give me those kind of answers, so I can check the research. But that's just me as a person. But I can see, okay, this is a good validated study, or this is like a very small study, so it's not very good advice, but that's something that I do. (PCP 4, NL) 2. That it can be wrong. There's been a handful of times that it will give me incorrect information, and even sometimes for my own knowledge to test it, I will ask it questions to answers, ask it questions for answers that I know and sometimes they'll give the wrong answer, but I know that I have learned what the correct answer is, so I will sometimes use it, but I always take a look at the articles that it cites, and sometimes try to take a look at those articles myself, to see whether or not it's a credible source. (PCP 11, US) |
|  | Importance of prompt writing and model input | 1. yes, it had some influence on my decision making. But in the same way I normally would look in on a website or something like that. But the way I approach these websites that changes because I ask my AI in perplexity in this case. I ask, for instance, what does the Nhg advise on, let's say vitamin D, or I experiment with it. I ask, I have a patient with a vitamin d of 30. She's 27 years old. What is, according to the Nhg. standard the dose of vitamin D I should give? But then I look it up because I want to check it. But I see most of the time, it's okay. But you have to ask a good problem, because if I just say, What dose should I use? Then it comes with American standards, so I have to ask for the Dutch situation. (PCP 3, NL) 2. Most of the time. I remember what kind of journal it was, so I tell the prompt to search in like "huisarts en wetenschap", for instance. in our case or the wtfg, and then it's more accurate defining it. But if you do not do it, then it gives you some general information from the Internet. (PCP 12, NL) |
|  |  |  |
| **Patient Safety and Data Security** | Concerns about data storage and breaches | 1. Yes, because you always have to be very aware that you don't use any patient information. And that's kind of a struggle, because i'm a CMIO. So I'm very aware of these kinds of things. But my staff like my yeah, my staff and my assistants. They aren't. So I have to really be explicit about how to use this kind of tools. And I think it's human nature, because it is so easy just to just upload a photo or upload a file which has patient information. So I think that's a very big yeah, not struggle, but I think that's a big data breach that we can obviously get by the simple tool. Yeah, it's such an easy tool. So the risk of a data breach is very likely. (PCP 4, NL) 2. But when, whenever colleagues are informed about it, besides enthusiasm, there's always this side note about, okay, but what happens with the data and how safe is this? And who's looking into those data. And I understand those questions. They're really, really important. It's health information, potentially very tricky privacy information. So yeah, I understand those concerns. And I think they need to be met. (PCP 14, NL) |
|  | Trust in Technology Providers | 1. And then there's, I think, a category of ethics around like the sort of beneficence and autonomy of the patient. Are you leaking or providing any information to a company, this large language model that it might use, or it might leak and I think that there have to be approaches and guardrails to set that up so that you're it's the appropriate use of the of the of the technology. (PCP 7, US) 2. It's all about big data and good AI, so that's something. Sometimes a thought I have like, how to keep it accessible, how to keep it working, even though a company goes bankrupt or got bought by some other company. So that's a bit of a worry I have sometimes. (PCP 9, NL) |
|  | De-identification and Anonymization | 1. I have to be extra careful, like making sure that you know all the information. It is hipaa compliant. And you know all the patient identifiers are removed. and we're just asking for just pure information. (PCP 2, US) 2. I don't know for sure. And for that reason I'm really careful about what I upload to the model, so no names, no birth dates and etcetera. (PCP 3, NL) |
|  | System Integration as a Security Barrier | 1. I think the biggest challenges, especially trying to use it within healthcare is definitely the concerns about security. And you know, patient information, that sort of aspect. And, I would say, it's not really integrated into our electronic medical record. So we're seeing all the information for a patient on one side and in one system. And if we want to try to incorporate ChatGPT, it's kind of completely separate and so that, I think is definitely a barrier until they figure out how to incorporate and make it safe and secure. (PCP 1, US) 2. So they have one with an integration into the EHR software we use. And there's a web browser version at the moment I push for the web browser one, because that's having more functionality in my situation, and is easier to grasp on in terms of security, because I can't really oversee how the integration fully works of the EHR. (PCP 9, NL) |
|  | Maintaining patient trust | 1. They may then start seeing physicians as just like technicians are. Maybe not a person who's gone through this much schooling to understand. You know how the human body works, and you know, as someone who really maybe knowledgeable and and kind of have sort of the intellect to figure out what's going on. And they may view. Physician says, Oh, look! You know, they're also just using this tool to figure out what's going on. (PCP 1, NL) 2. I think the other ethical issue that I guess I could think about has more to do with like conflict with patients being uncomfortable with it. And you know what to do in the instance that a patient declines, or to having an AI tool being utilized as part of their visit, and whether we can create the ability to be responsive to that or not. (PCP 5, US) |
|  | Overreliance | 1. Recommendations made that harm a patient, or lead to a patient getting inappropriate or inadequate care. That's kind of the biggest thing, the risk of patient safety for that patient themselves. (PCP 5, US) 2. Yeah, I don't validate it. I'll just take my advances from it and move on. (PCP 8, NL) |

1. Braun V, Clarke V. Using thematic analysis in psychology. Qual Res Psychol. 2006;3(2):77–101.
